# COVID-19 in German Nursing Homes: A Panel Study on Morbidity, Burden of Care, and Social Isolation

**DOI:** 10.1093/geroni/igab046.1574

**Published:** 2021-12-17

**Authors:** Kathrin Seibert, Dominik Domhoff, Franziska Heinze, Benedikt Preuss, Heinz Rothgang, Karin Wolf-Ostermann

**Affiliations:** 1 Institute for Public Health and Nursing Research, Universtity of Bremen, Bremen, Germany; 2 Institute for Public Health and Nursing Research, University of Bremen, Bremen, Germany; 3 Research Center on Inequality and Social Policy (SOCIUM), university of Bremen, Bremen, Germany; 4 Research Center on Inequality and Social Policy (SOCIUM), University of Bremen, Bremen, Germany; 5 University of Bremen, University of Bremen, Bremen, Germany

## Abstract

Germany was hit by the second wave of the pandemic much harder than by the first wave. The study aims to describe the extent to which nursing homes (NH) are affected by COVID-19. About 8,000 NHs were invited to participate in two waves of an online survey, with a share of 5-10% participating. The share of all deceased NH-residents with COVID-19 is about 50% (04/2020-02/2021). Factors that increase the risk of an outbreak in NH are the spread of the virus in the population, the size of the institution and staff-resident-ratio. The initial lack of protective equipment has decreased during the second wave, but the facilities have to cope with massive additional care needs with reduced staff. NHs have partly banned contacts between residents and relatives. As a conclusion the support of NH in their attempt to fight the impact of this and further pandemic situations requires highest attention.

